# Influence of Embedding SMA Fibres and SMA Fibre Surface Modification on the Mechanical Performance of BFRP Composite Laminates

**DOI:** 10.3390/ma11010070

**Published:** 2018-01-04

**Authors:** Yanfei Liu, Zhenqing Wang, Hao Li, Min Sun, Fangxin Wang, Bingjie Chen

**Affiliations:** 1 College of Aerospace and Civil Engineering, Harbin Engineering University, Harbin 150001, China; yanfeiliu@hrbeu.edu.cn (Y.L.); lihao0202@hrbeu.edu.cn (H.L.); sunmin@hrbeu.edu.cn (M.S.); wangfangxin@hrbeu.edu.cn (F.W.); 2 School of Mechanical Engineering, University of Leeds, Leeds LS2 9JT, UK; bn16b3c@leeds.ac.uk

**Keywords:** polymer composites, basalt fibre, SMAHCs, surface modification

## Abstract

In this paper, a new shape memory alloy (SMA) hybrid basalt fibre reinforced polymer (BFRP) composite laminate was fabricated and a new surface modification method with both silane coupling agent KH550 and Al_2_O_3_ nanoparticles was conducted to enhance the interface performance. The mechanical performance of BFRP composite laminates with and without SMA fibres and the influence of SMA surface modification were studied in this paper. Different SMA fibre surface treatment methods, including etching with both H_2_SO_4_ and NaOH, modification with the silane coupling agent KH550 and new modification method with both KH550 and Al_2_O_3_ nanoparticles, were conducted to enhance the bonding between the SMA fibres and polymer matrix. Scanning electron microscopy (SEM) was used to observe the micromorphology of the SMA fibre surfaces exposed to different treatments and the damage morphology of composite laminates. The mechanical performance of the composites was investigated with tensile, three-point bending and low-velocity impact tests to study the influence of embedded SMA fibres and the different surface modifications of the SMA fibres. The results demonstrated that the embedded Ni-Ti SMA fibres can significantly enhance the mechanical performance of BFRP composite laminates. SMA fibres modified with both the silane coupling agent KH550 and Al_2_O_3_ nanoparticles illustrate the best mechanical performance among all samples.

## 1. Introduction

As environmentally friendly natural fibre, basalt fibre have attracted attention ever since they were discovered in 1923 [1]. Compared to glass fibre, which is commonly used in industry, basalt fibre shows a higher elastic modulus, tensile strength and elongation of fracture [2,3]. Comparing to carbon fibre, basalt fibre have higher toughness while the price is much lower. In addition to the good mechanical performance, basalt fibre also shows great corrosion resistance to alkaline environment at the same time [4]. Basalt fibres can be used as reinforcement material to enhance the performance of concrete structure owing to its high performance and environmental friendly property [5,6]. In recent years, due to their excellent performance, researchers have incorporated basalt fibre, as reinforcement material, into polymer matrices to fabricate basalt/polymer composites. To investigate the mechanical performance of basalt fibre reinforced polymer composites, Lopresto et al. [7] compared the mechanical performance of composite laminates reinforced with basalt fibre and glass fibre. The experimental results show that the basalt/polymer composite laminates have a higher elastic modulus, tensile strength and flexural strength than the glass/polymer composites. In addition, the basalt/polymer composites also have better impact resistance performance. They also investigated the performance of the interface between the basalt fibres and polymer matrix through short beam strength tests, which was proven to be similar to that between glass fibres and the polymer matrix. De Rosa et al. [8] investigated the post-impact behaviour of glass fibre-reinforced polymer (GFRP) composite laminates and basalt fibre-reinforced polymer (BFRP) composite laminates. The post-impact properties of the BFRP composite laminates were slightly superior to the properties of the GFRP composite laminates. All of the above studies prove that basalt fibre is an ideal alternative to glass fibre. Wei et al. [9] investigated the seawater corrosion resistance properties of a BFRP composite and compared the material to a traditional GFRP composite. Researcher have also attempted to use basalt fibre along with other fibres to fabricate hybrid composites. Sarasini et al. [10] and Dehkordi et al. [11] studied the performance of a glass/basalt woven fabric/epoxy resin composite and basalt/nylon woven inter-ply hybrid composite under low-velocity impact. Basalt fibres were also combined with flax, hemp, glass, carbon, jute and Kevlar fibres to investigate the mechanical performance of different hybrid composites [12,13,14,15,16,17,18,19]. Fiore et al. [20] and Dhand et al. [21] reviewed previous studies on basalt fibre, which further prove that it is an ideal reinforce material owing to its excellent mechanical, thermal and chemical performance.

Shape memory alloys (SMAs), as a unique material with specific performances, including shape memory effects, superelasticity and pseudoelasticity, have been used in the aerospace industry and medical industry for decades. In recent years, due to their properties, SMA fibres have been used by researchers to fabricate shape memory alloy hybrid composite (SMAHC) laminates with enhanced mechanical performance. Lei et al. [22] compared the tensile strength of GFRP and glass fibre/SMA fibre hybrid composites. They found that the addition of SMA fibres can significantly improve the tensile strength of the composite laminates. Zhou et al. [23] utilized the shape memory effect to fabricate smart composite beams and tested the mechanical performance with single-cycle and multi-cycle bending experiments. As the composite laminates have weak through-the-thickness performance, researchers have embedded SMAs into composite laminates to enhance the impact resistance due to the good energy absorption performance of SMAs. Tsoi et al. [24] and Meo et al. [25] investigated the mechanical performance under low-velocity impact. In their studies, it was found that the addition of SMA fibres enhanced the impact resistance of the SMAHC.

The compatibility between different materials also have influence on the performance of composite. Flory-Huggins interaction parameter was used to investigate the compatibility of polymer blends [26,27,28]. The thermal, mechanical and morphological properties of different polymer blends were investigated considering the compatibility between different constituent [29,30,31,32]. In the study of Lei et al. [22], it was found that the poor interface bonding capacity between the SMA fibres and polymer matrix leads to a decrease in the mechanical performance of the SMAHC. To solve this problem, researchers have proposed many methods to modify the surface of SMA fibres to enhance the bonding between the SMA fibres and polymer matrix. Nitinol fibres were treated with acid solution by Huang et al. [33] and Jang et al. [34], which enhanced the surface roughness of the SMA fibres. Neuking et al. [35] and Rossi et al. [36] treated Ni-Ti fibres with different combinations of physical and chemical treatments, including rinsing with acetone, mechanical grinding and polishing, acid corrosion, alkali corrosion and electro polishing. Laser etching [37,38,39] has been used by researchers to enhance the surface roughness of the Ni-Ti surface and laser gas nitriding (LGN) [40,41,42] has also been used to enhance the interface performance. It was found that an oxide layer formed on the Ni-Ti surface due to laser etching, while a TiN layer formed on the Ni-Ti surface due to LGN treatment. In our previous research [43], SMA fibres were modified with silane coupling agent and Al_2_O_3_ nanoparticles to achieve better bonding performance with polymer matrix. The method was proven to have significant effect on enhancing the adhesion strength in a single fibre pull-out experiment.

In this paper, basalt fibre/SMA/polymer composite laminates were fabricated to enhance the performance of BFRP composite laminates, especially the through-the-thickness performance, which was never investigated. In addition, considering that the weak interface performance between SMA fibres and polymer matrix will reduce the overall performance of composite laminates but there are limited research focusing on that, we use some traditional methods and a new surface modification method with both silane coupling agent KH550 and Al_2_O_3_ nanoparticles to modify the interface thus obtaining a better overall performance of composite laminates.

## 2. Experimental Details

### 2.1. Materials and Manufacturing

In this study, all reagents were used as received. Vinyl ester resin is widely used in industry for its relative good mechanical performance, corrosion resistance performance and low price. In our study, because of the good performance as well as the characteristics of easy processing, DERAKANE^®^ (Ashland Inc., Lexington, KY, USA) 411-350 vinyl ester resin was used as the matrix of the composite laminates. Methyl ethyl ketone peroxide (MEKP) and dimethylaniline were used as a hardening agent and accelerating agent, respectively. The polymer, hardening agent and accelerating agent were purchased from Ashland Inc., Lexington, KY, USA. The weight ratio of the vinyl ester resin, hardening agent and accelerating agent is 100:1:0.25, which ensures that the polymer matrix can be cured at room temperature without additional treatment. The tensile strength, tensile modulus, flexural strength and flexural modulus of the vinyl ester resin matrix are 86 MPa, 3.2 GPa, 150 MPa and 3.4 GPa, respectively. The gelation time is approximately 1.5 h, which ensures that the reinforcement materials are fully soaked by the polymer matrix. The silane coupling agent used in this study is analytically pure 3-triethoxysilylpropylamine APTES (KH550), which was purchased from Energy Chemical, Shanghai, China. Unidirectional basalt fibre cloth (provided by Tongxiang Mengtai Reinforced Composite Material Company, Tongxiang, China) was used as a reinforced material in the composite laminates. The tensile strength, modulus and elongation rate of basalt fibre cloth are 3080 GPa, 95 GPa and 3.1%, respectively while the thickness and surface density of single fibre cloth are 0.2 mm and 280 g/m^2^, respective. Commercial SMA fibres were provided by Jiangyin Fasten-PLT Materials Science Co., Jiangyin, China. The Al_2_O_3_ nanoparticles used in our study have an average diameter of 100 nm. The diameter of the SMA fibres is 0.5 mm; the weight ratio of Ni is 51.8% and the austenite finish temperature (A_f_) of the SMA fibres is 15 °C. The ultimate tensile strength of the Ni-Ti fibres is 1226 MPa. The as-received Ni-Ti SMA fibres were coated with a thin oxide layer. Before use, all fibres were cleaned with degreased gauze soaked in acetone to remove impurities adhered on the surface of the Ni-Ti fibres.

**Figure 1:**
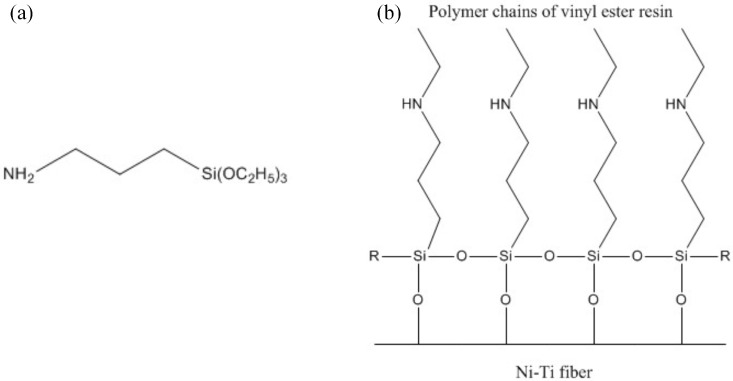
(**a**) Chemical structure of the silane coupling agent KH550 and (**b**) schematic diagram of the effective mechanism of the silane coupling agent KH550.

To investigate the influence of Ni-Ti surface treatment on the overall performance of the composite laminates, the Ni-Ti fibres were divided into four groups. The first group of Ni-Ti fibres was only cleaned with degreased gauze soaked in acetone. The second group of Ni-Ti fibres was first cleaned using the same procedure as the first group of fibres. Then, the second-group fibres were immersed in concentrated H_2_SO_4_ for 20 min with sonication. Then, the Ni-Ti fibres were washed by immersion in acetone and hexane for 20 min each with sonication. After that, to increase the concentration of hydroxides on the Ni-Ti fibre surface, the fibres were immersed in a 1 M NaOH solution for 20 min. Finally, the Ni-Ti fibres were immersed in concentrated H_2_SO_4_ for 5 min with sonication to remove residual sodium ions on the fibre surface. After that, the fibres were washed with deionized water and dried for future use. The third group of Ni-Ti fibres was firstly treated with H_2_SO_4_ and NaOH solution, the same as the second group, as pre-treatment. Then, the treated Ni-Ti fibres were immersed in a dry toluene solution with 3 wt % silane coupling agent KH550 for 6 h at room temperature, then dried and stored under nitrogen for future use. The chemical structure of the silane coupling agent KH550 and the schematic diagram of the effective mechanism of KH550 are shown in Figure 1. The polymer chains of the vinyl ester resin can be connected to the hydroxyl groups through the action of KH550.

The fourth group of Ni-Ti fibres was first treated with H_2_SO_4_ and NaOH solution as pre-treatment—the same as the second group—then immersed in a dry toluene solution containing 3 wt % silane coupling agent KH550 and 3 wt % Al_2_O_3_ nanoparticles, which was proven by Wang et al. [43] to have the best effect on enhancing the performance of the interface between the Ni-Ti fibres and polymer matrix. The treated fibres were dried and stored under nitrogen for future use.

**Figure 2:**
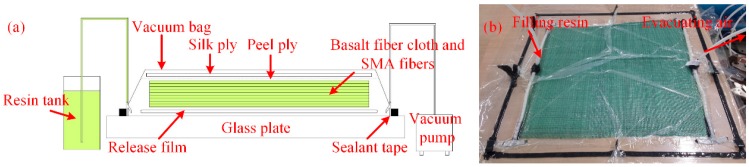
Fabrication of the composite laminates via the VARTM method. (**a**) Schematic diagram and (**b**) picture of the real object.

In this paper, composite laminates with and without SMA fibres were fabricated to investigate the influence of embedded SMA fibres on the overall mechanical performance of the SMAHCs. SMAHC laminates embedded with SMA fibres exposed to different treatments were also fabricated to investigate the influence of Ni-Ti surface modification on the mechanical performance of the SMAHCs. Composite laminates without SMA fibres were embedded with 8-ply unidirectional basalt fibre cloth with a stacking sequence of [0°/0°/90°/90°/0°/0°/90°/90°]. Depending on the experiment, the SMAHC laminates were produced with different fibre stacking sequences. For the tensile tests, the stacking sequence was [0°/0°/90°/90°/SMA 0°/0°/0°/90°/90°] to give the specimens better symmetry. For the three-point bending and low-velocity impact tests, the stacking sequence was [0°/0°/90°/90°/0°/0°/90°/SMA 90°/90°], because in the study of Tsoi et al. [24], SMA fibres embedded in the lower part of the composite laminates had a better effect on enhancing the low-velocity impact resistance. Meanwhile, SMA fibres embedded in the lower part of the composite laminate can also undergo maximum deformation in the three-point bending test, which means that the performance of the SMA fibres could be better utilized. According to the different dimensions, the amount of embedded Ni-Ti fibres in the specimens for the tensile, three-point bending and low-velocity impact tests were 10, 5 and 13, respectively. All Ni-Ti SMA fibres were aligned parallel in the centre of the specimens with a gap of 2 mm. The composite laminates were fabricated via vacuum-assisted resin transfer moulding (VARTM). The schematic diagram of the VARTM method is shown in Figure 2.

The composite laminates were cured at room temperature for 24 h for hardening and then cut into small pieces with a diamond slitting wheel, according to the standards of the tensile, three-point bending and low-velocity impact tests. The dimensions of the specimens used in the tensile test, three-point bending test and low-velocity impact test were 200 × 25 × 1.7 mm, 69 × 13 × 1.8 mm and 100 × 100 × 1.8 mm, respectively. The volume fraction of Ni-Ti SMA fibres in the tensile, three-point bending and low-velocity impact specimens were 4.62%, 4.19% and 1.42%, respectively. All the types of specimens were summarized in Table 1. The test specimens were dried in a vacuum oven for 24 h at 60 °C to eliminate the influence of water in the specimens. The specimens were stored in a dry container for future tests.

### 2.2. Tensile Test Setups

**Figure 3:**
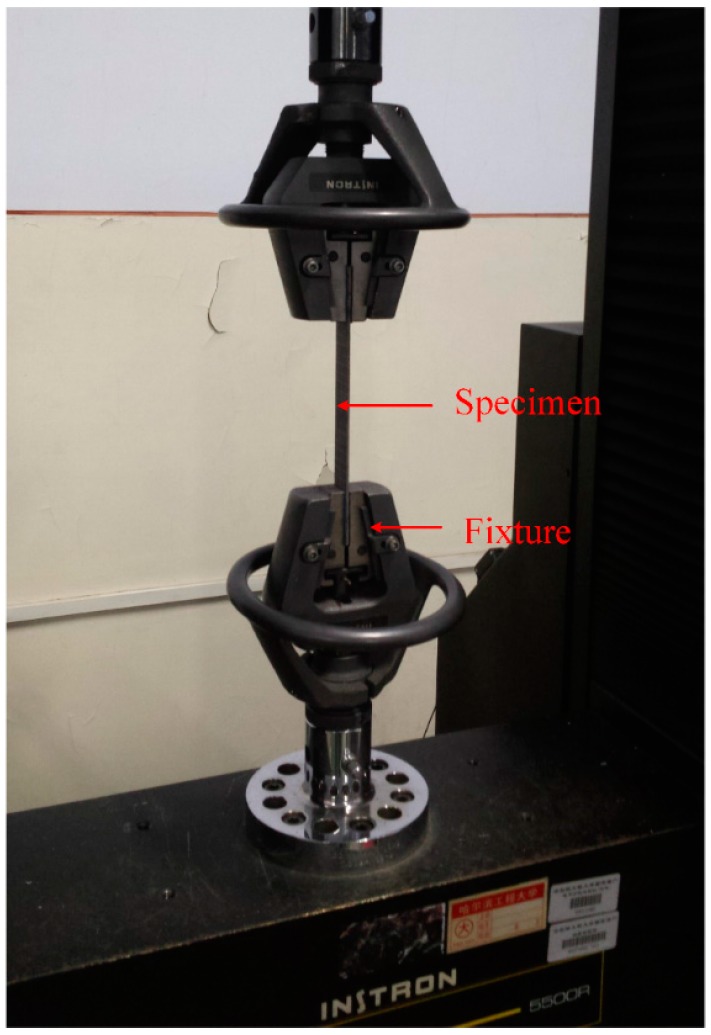
Testing setup of the tensile test.

Tensile tests were performed accordance to the ASTM D7264/D7264M-07 standard [44]. In all the tests, each group have 5 specimens for more accurate results. All uniaxial tensile tests of the composite laminates were conducted with an INSTRON 4505 servo-electric testing machine at a cross-head speed of 2 mm/min, as shown in Figure 3. The calibrating length in this study is 100 mm.

### 2.3. Three-Point Bending Test Setups

**Figure 4:**
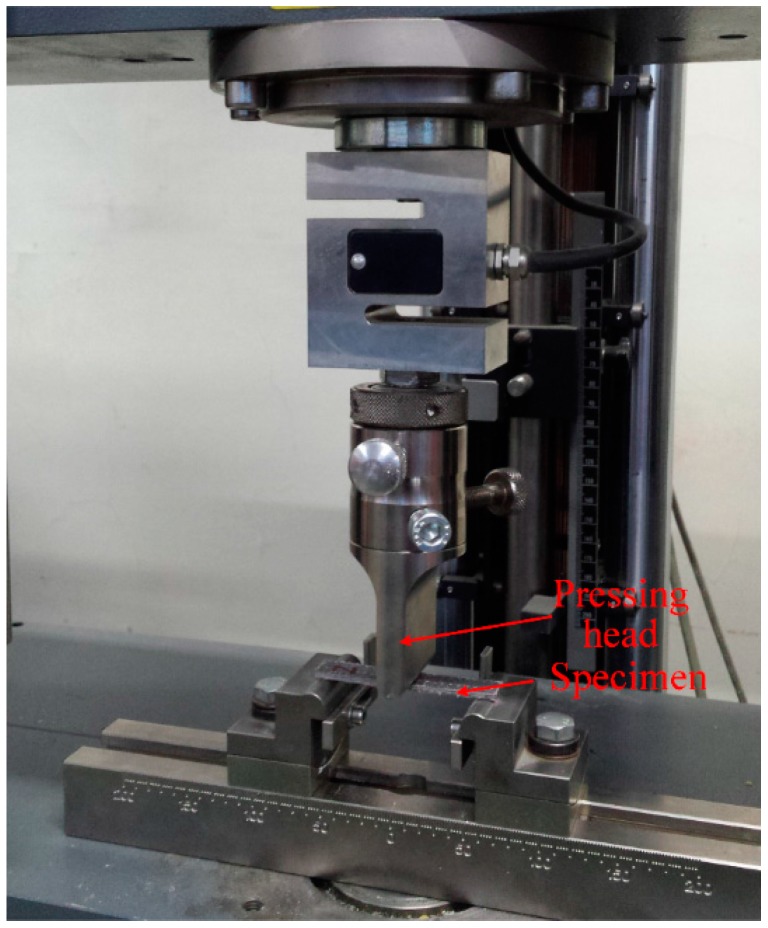
Testing setup of the three-point bending test.

Three-point bending tests were conducted accordance to the ASTM D3039/D3039M-07 standard [45], with a Zwick-Roell Z010 servo-electric testing machine (Zwick Roell, Ulm, Germany). The span length was 57.6 mm and the cross-head speed was 1 mm/min. The testing setup of the three-point bending test is shown in Figure 4.

### 2.4. Drop-Weight Low-Velocity Impact Test Setup

**Figure 5:**
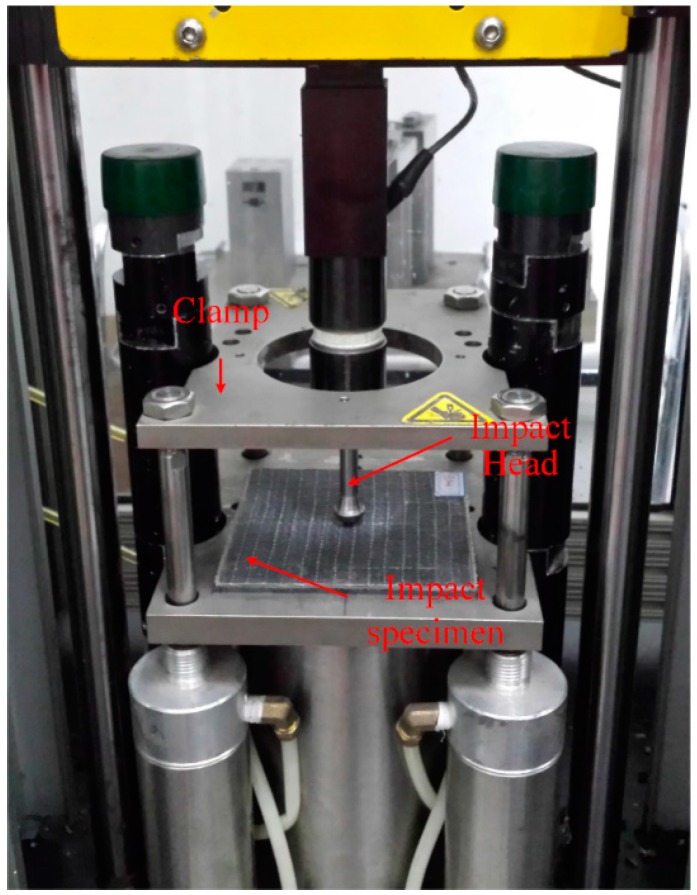
Testing setup of the low-velocity impact test.

Low-velocity impact tests were conducted accordance to the ASTM D7136/D7136M-05 standard [46], using a PC-driven Instron Dynatup 9250 HV (Instron, Norwood, MA, USA) pneumatic-assisted drop-weight impact tester, which has a pneumatic brake to avoid multiple strikes, as shown in Figure 5.

Before the impact test began, to avoid movement of the test specimen, the specimen was clamped by the testing machine. The mass of the impact head was 4.0496 kg. The drop height was set according to the impact energy, which was pre-set to 24 J in this study. The parameters in the impact tests, including the impact velocity and impact force, were measured by the photodiodes of the impact machine and recorded in the system.

## 3. Results and Discussion

### 3.1. Micromorphology Analysis

**Figure 6:**
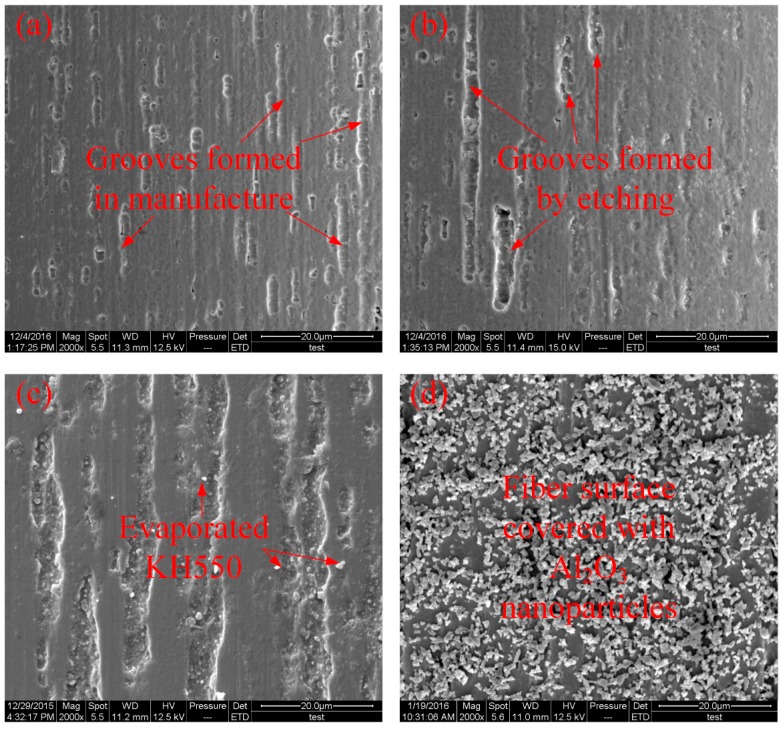
Surface micromorphology of the Ni-Ti fibres (**a**) without treatment; (**b**) etched with H_2_SO_4_ and NaOH; (**c**) modified with the silane coupling agent KH550 and (**d**) modified with both the silane coupling agent KH550 and Al_2_O_3_ nanoparticles.

The bonding capacity between the Ni-Ti SMA fibres and polymer matrix is influenced by surface modification method. Different treatments lead to different surface roughness. The micromorphology of the Ni-Ti SMA fibres surface was observed via scanning electron microscopy (SEM) (FEI QUANTA 200, FEI Inc., Hillsboro, OR, USA). Figure 6a–d shows the micromorphology of SMA fibres that were untreated, treated with H_2_SO_4_ and NaOH solution, modified with the silane coupling agent KH550 and modified with both KH550 and Al_2_O_3_ nanoparticles, respectively.

In Figure 6a, it can be seen that the untreated Ni-Ti fibres have a number of grooves on the fibre surface. The grooves are assumed to be produced during the manufacturing process. In Figure 6b, it can be observed that some of the grooves on the surface of the corroded Ni-Ti fibre are deeper than those in the untreated Ni-Ti fibre. However, the corrosion is not severe due to the relatively short corrosion time. For the fibre modified by the silane coupling agent KH550, it can be observed in Figure 6c that there are many small spots on the fibre surface, which are due to the evaporation of the silane coupling agent KH550. Meanwhile, the depth of the grooves on the fibre surface show no difference from those in the corroded sample, which proves that treatment with KH550 does limited damage to the Ni-Ti fibre. In Figure 6d, many well-dispersed Al_2_O_3_ nanoparticles are observed to be attached to the surface of the Ni-Ti fibres. Wang et al. [43] noted that the addition of Al_2_O_3_ nanoparticles can significantly enhance the roughness of the composite interface and the addition of the silane coupling agent KH550 can improve the dispersion of the nanoparticles to reduce the amount of initial imperfections in the composite.

**Figure 7:**
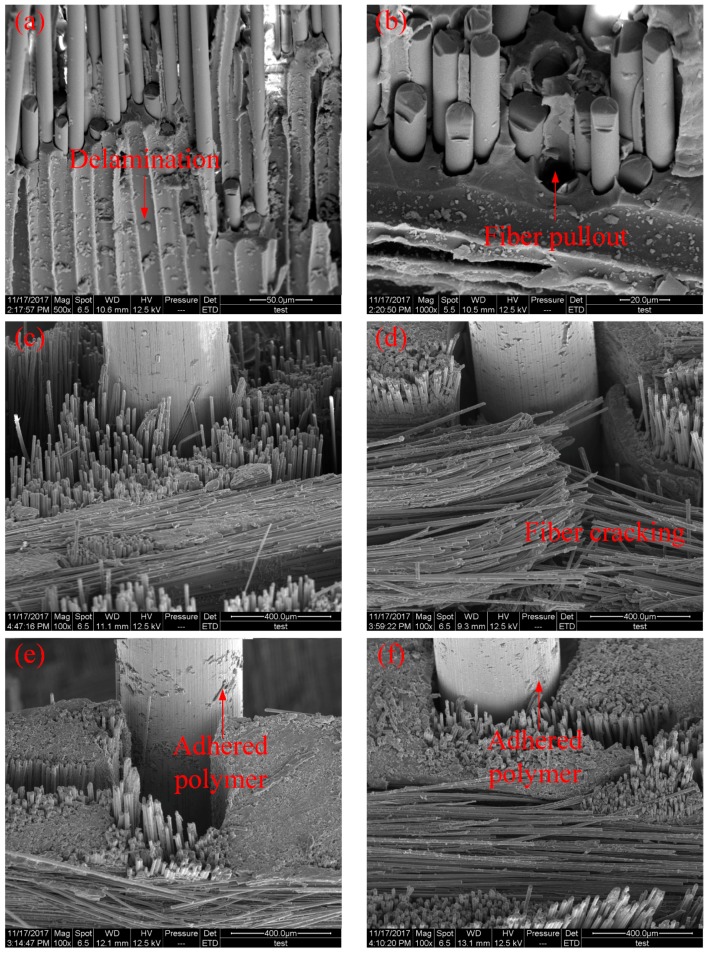
Micromorphology of specimens under low-velocity impact tests. (**a**,**b**) Without SMA fibres; (**c**) embedded SMA fibres without treatment; (**d**) etched with H_2_SO_4_ and NaOH; (**e**) modified with the silane coupling agent KH550 and (**f**) modified with both the silane coupling agent KH550 and Al_2_O_3_ nanoparticles.

In this study, tested low-velocity impact specimens were also scanned with SEM to observe the inner failure of laminates and to illustrate the effect of SMA fibres and SMA fibre surface modification in the hybrid composite system. Figure 7a–f shows the damaged specimens under low-velocity impact tests. Figure 7a,b shows the failure morphology of BFRP laminate without SMA fibres. All types of failures can be observed, including delamination, matrix cracking, fibre fracture and interface debonding between basalt fibres and polymer matrix. In Figure 7c–f, specimens embedded with SMA fibres unmodified, etched, modified with KH550, modified with both KH550 and nanoparticles were observed. It can be seen that to the unmodified SMA fibres, when the debonding occurs between fibres and polymer matrix, no polymer is adhered on SMA fibres, which indicate the poor bonding capacity. To the modified SMA fibres, especially SMA fibres modified by KH550 and both KH550 and nanoparticles, more polymer matrix can be observed on the fibre surface, proving that modified SMA fibres have better bonding capacity in the composite system, which is consistent with our earlier studies [43].

### 3.2. Influence of Embedding SMA Fibres and Surface Modification on Tensile Performance

**Figure 8:**
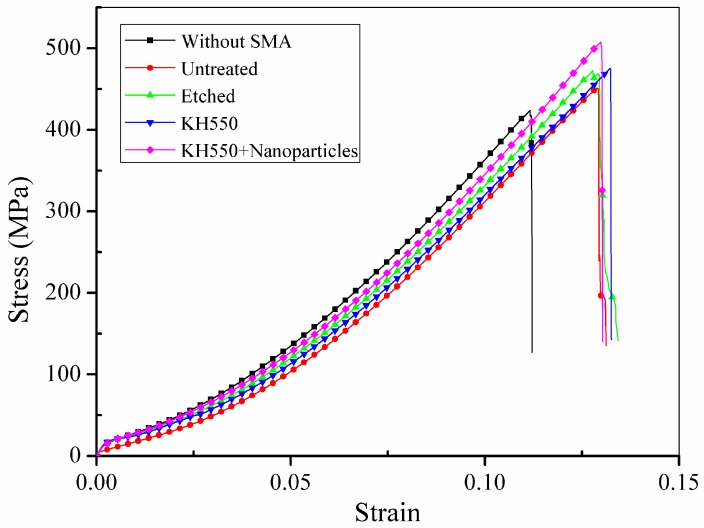
Representative stress-strain curves of the uniaxial tensile tests.

The tensile performance of the composite laminates was examined under uniaxial tensile tests. Representative stress-strain curves of the BFRP laminates without Ni-Ti SMA fibres and with embedded Ni-Ti SMA fibres exposed to different treatments are shown in Figure 8. The black line represents the BFRP laminates without Ni-Ti SMA fibres. The red line, green line, blue line and purple line represent the stress-strain curves of the BFRP laminates embedded with Ni-Ti SMA fibres that were untreated, etched with H_2_SO_4_ and NaOH, modified with 3% KH550 and modified with both 3% KH550 and 3% Al_2_O_3_ nanoparticles, respectively.

**Figure 9:**
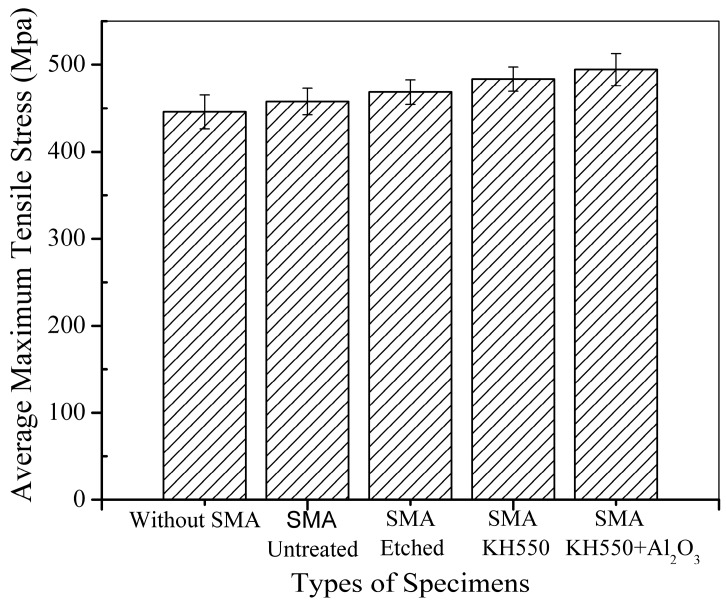
Average maximum tensile stress of the different samples.

It can be observed that all BFRP laminates embedded with Ni-Ti SMA fibres show higher tensile strength than the BFRP laminate without Ni-Ti SMA fibres because of the high tensile strength of the Ni-Ti SMA fibres. However, it can be noticed that compared to samples embedded with modified Ni-Ti fibres, the BFRP laminate embedded with untreated Ni-Ti fibres shows limited enhancement compared to the BFRP laminate without Ni-Ti fibres. The average maximum tensile stress of the different samples is shown in Figure 9.

Although the Ni-Ti fibres have a high tensile strength of 1226 MPa, which is much higher than the tensile strength of the BFRP laminate, the BFRP laminate embedded with untreated fibres had only a 2.66% increase in tensile strength. This could be attributed to poor adhesion between the Ni-Ti SMA fibres and polymer matrix, which was also found by Lei et al. [22]. In the laminates embedded with treated Ni-Ti SMA fibres, the tensile strength significantly increased. Laminates with Ni-Ti SMA fibres treated with H_2_SO_4_ and NaOH solution, modified with the silane coupling agent KH550 and modified with both KH550 and Al_2_O_3_ nanoparticles showed 5.10%, 8.43% and 10.91% enhancement, respectively, compared to the BFRP laminate without SMA fibres. Different treatments have different mechanisms of enhancing the performance of the interface between the Ni-Ti SMA fibres and polymer matrix. In the SMA fibres etched with H_2_SO_4_ and NaOH solution, there are many deep ravines on the surface of the Ni-Ti fibres compared to the untreated fibres, as seen in Figure 6. The enhanced surface roughness of the Ni-Ti fibres improve the mechanical bonding between the Ni-Ti fibres and polymer matrix. When a tensile force is applied, the stress can be effectively transferred from the polymer matrix to the Ni-Ti fibres, which leads to an enhancement in the tensile strength of the laminate. For the BFRP laminate embedded with Ni-Ti fibres modified with KH550, the mechanism is different. The surface roughness of the Ni-Ti fibres modified with the silane coupling agent KH550 showed no difference from the fibres processed with H_2_SO_4_ and NaOH solutions; however, the tensile strength of the laminate was enhanced. The addition of the silane coupling agent KH550 improved the compatibility between the inorganic Ni-Ti fibres and organic polymer matrix through the coupling action. The polymer chains were connected to the hydroxyl units on the surface of the Ni-Ti fibres, which improved the bonding capacity as well as enhanced the surface roughness, leading to an increase in the tensile strength of the laminate. The laminate embedded with Ni-Ti fibres modified with both the silane coupling agent KH550 and Al_2_O_3_ nanoparticles showed the best performance among all samples. Wetzel investigated the influence of adding nanoparticles into epoxy resin [47]. The presence of nanoparticles can lead to crack deflection in the epoxy resin matrix. The plastic deformation of the composites is also influenced by the addition of nanoparticles. Debonding between the nanoparticles and epoxy matrix leads to energy dissipation, which enhances the toughness of the composites. In the tensile test of the BFRP laminate embedded with Ni-Ti SMA fibres modified with KH550 and Al_2_O_3_ nanoparticles, the reinforcing mechanism can be described as follows. First, the addition of the silane coupling agent KH550 enhanced the chemical bonding between the Ni-Ti fibres and polymer matrix. KH550 also enhanced the dispersion of Al_2_O_3_ nanoparticles on the Ni-Ti fibre surface, which reduced particle clustering and initial imperfections at the interface between the Ni-Ti fibres and polymer matrix. Cracks in the composite during tensile testing usually propagate along the weak interface. The addition of Al_2_O_3_ nanoparticles can lead to crack deflection at the interface, which can limit the extension of cracks along the interface. When a crack develops along the interface, debonding between the nanoparticles and matrix leads to energy dissipation, which can also prevent breakage of the composite laminate. Nanoparticles can also enhance the roughness of the interface between the Ni-Ti fibres and matrix, which can improve mechanical occlusion between the Ni-Ti fibres and polymer matrix. Stress in the composites can better transfer from the matrix to the embedded reinforcement fibres, which that ensures the high tensile strength of the Ni-Ti fibres is better reflected.

### 3.3. Influence of Embedding SMA Fibres and Surface Modification on Flexural Performance

**Figure 10:**
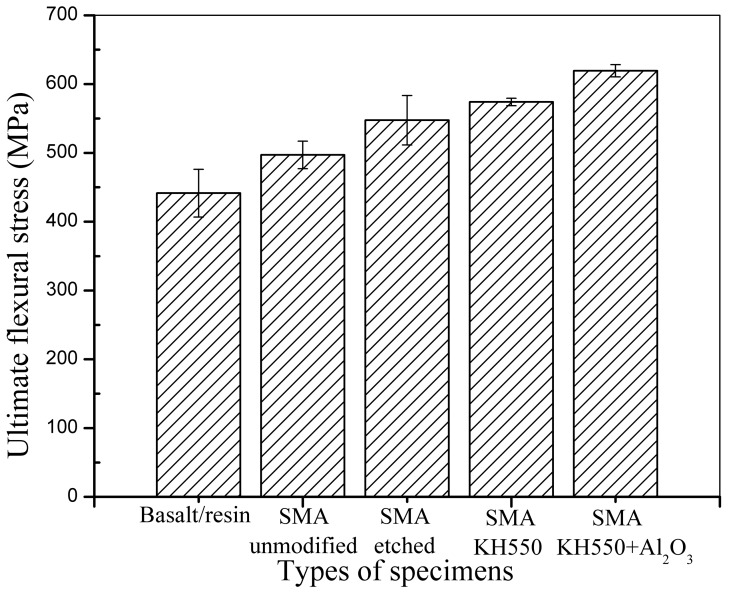
Average ultimate flexural stress of the different samples.

The flexural performance of the composite laminates was examined under three-point bending tests. In addition to the effect of the embedded SMA fibres on the flexural performance of the SMA hybrid BFRP composite, the influence of SMA surface modification on the overall flexural performance of the composites was also investigated. The average ultimate flexural stresses of the BFRP laminates without Ni-Ti SMA fibres and with embedded Ni-Ti SMA fibres exposed to different treatments are shown in Figure 10.

It can be seen that the addition of SMA fibres in the SMAHC laminate can significantly enhance the flexural strength. Although the SMA volume fractions of the specimens used in the tensile and three-point bending tests are similar, the enhancement effect of the SMA fibres in the specimens for the three-point bending tests is better than that in tensile specimens. This can be attributed to the location of the SMA fibres in the SMAHC laminate beams. When subjected to a flexural load, the lower part of the bent specimen would undergo maximum deformation. That is, SMA fibres embedded in the lower part of the beam can better dissipate stress during the bending process, which allows their excellent mechanical performance to be better exploited. Comparing the test results of the specimens embedded with SMA fibres, it can be seen that the specimen embedded with unmodified SMA fibres has the lowest enhancement among all specimens, which can be attributed to poor bonding between the SMA fibres and polymer matrix. SMA fibres processed with H_2_SO_4_ and NaOH have a higher surface roughness than the unmodified SMA fibres, which can enhance mechanical bonding between the fibres and matrix. However, the poor affinity between the SMA fibres and polymer matrix did not change upon treatment with H_2_SO_4_ and NaOH. The specimen with SMA fibres modified with the silane coupling agent KH550 shows better chemical bonding between the SMA fibres and polymer matrix, while the specimen with SMA fibres processed with both KH550 and Al_2_O_3_ nanoparticles show both better chemical bonding and more surface roughness, as the presence of Al_2_O_3_ nanoparticles can also reduce crack extension along the interface. Specimens embedded with SMA fibres modified with KH550 or both KH550 and Al_2_O_3_ nanoparticles have better flexural strength because of the enhanced chemical bonding between SMA fibres and polymer matrix. Specimen with SMA fibres modified with both KH550 and Al_2_O_3_ have the best flexural strength among all the bending specimens because chemical bonding and surface roughness were both enhanced.

### 3.4. Influence of Embedding SMA Fibres and Surface Modification on Impact Resistance Performance

**Figure 11:**
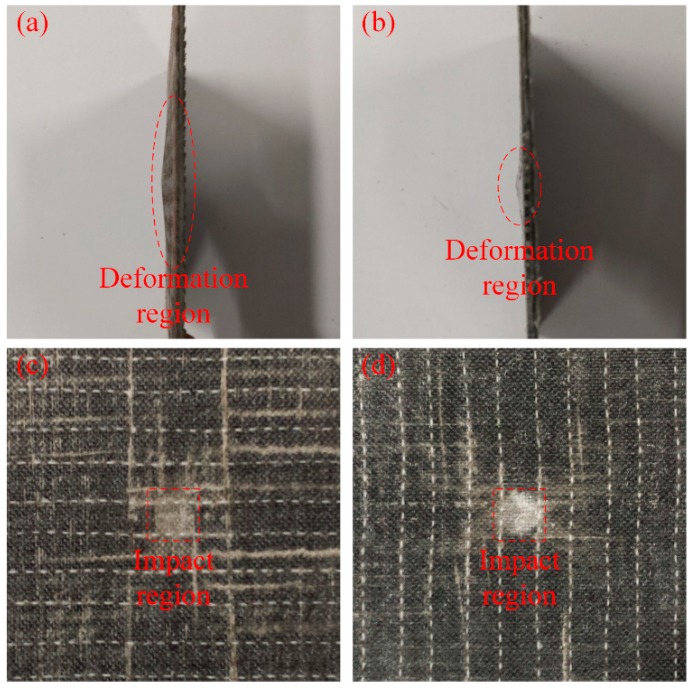
Damage morphology of the specimens: (**a**) side view of a damaged specimen without SMA fibres; (**b**) side view of a damaged specimen with SMA fibres; (**c**) front view of a damaged specimen without SMA fibres and (**d**) front view of a damaged specimen with SMA fibres.

The damage morphologies of the impact specimens with and without SMA fibres are compared in Figure 11.

**Figure 12:**
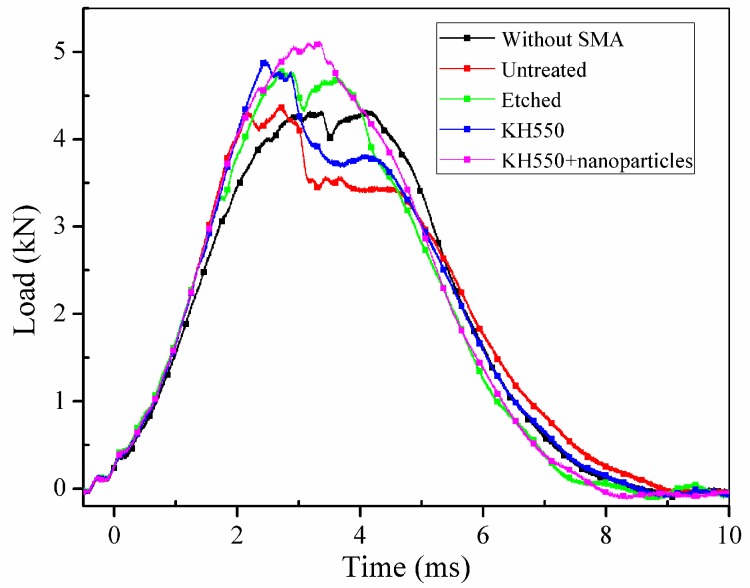
Time-contact load curves of different specimens.

**Figure 13:**
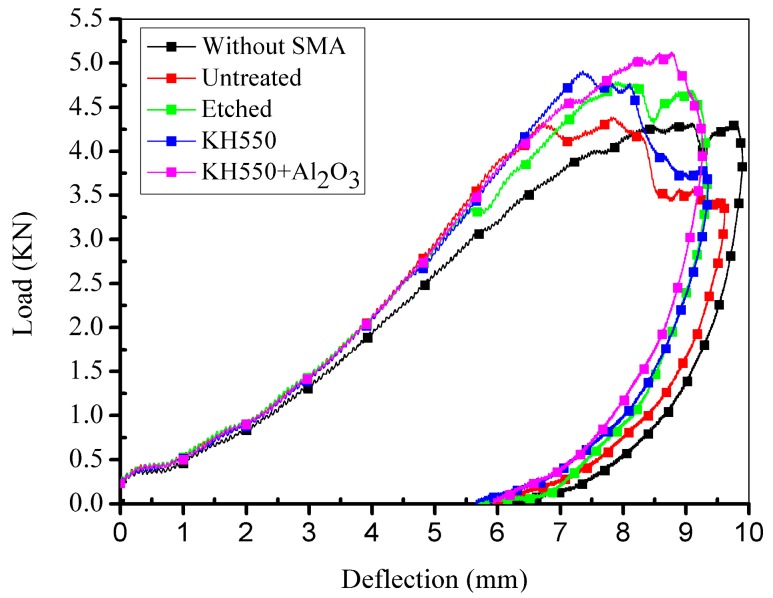
Deflection-contact load curves of different specimens.

**Figure 14:**
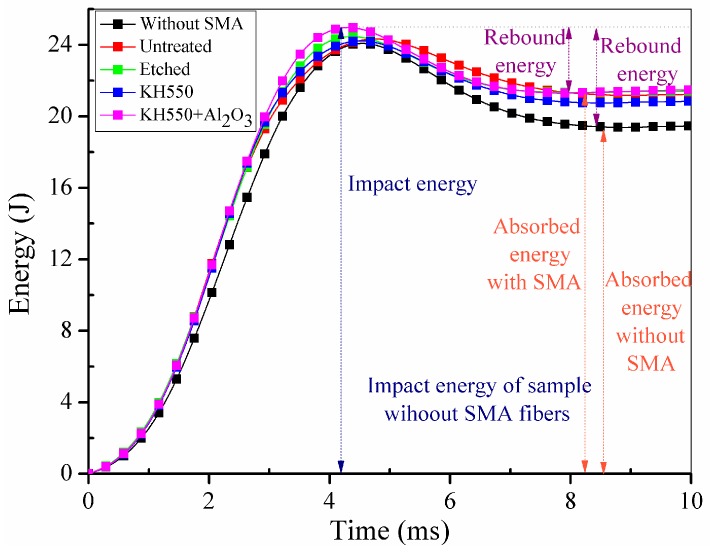
Time-energy curves of different specimens.

Figure 11a,b illustrates the side view of the damaged specimens without and with SMA fibres, respectively. Under the same impact energy, it can be seen that the damage region of specimens without SMA fibres is larger than specimens embedded with SMA fibres. Figure 11c,d illustrates the front view of the damaged specimens without and with SMA fibres, respectively. It is clearly that the surface damage of the specimen with SMA fibres is more severe compared with the specimen without SMA fibres, which can be attributed to the relatively high stiffness of the specimen embedded with SMA fibres. Cantwell and Morton [48] found that impact damage of a laminate with low stiffness occurs in the lower part while impact damage of a laminate with higher stiffness occurs in the upper part, which is consistent with our observations. By investigating the damage morphology of specimens without and with SMA fibres, it can be concluded that the damage of composite laminate under low-velocity impact can be restrained by embedded SMA fibres. The typical damage types of SMA/basalt fibre/polymer composite laminates under low-velocity impact are matrix cracking, basalt fibre cracking, debonding between basalt fibres and polymer matrix and debonding between SMA fibres and polymer matrix. The damage evolution in composite laminates can be described as follows. Micro cracking firstly occurred in the polymer matrix. The cracks would extend along the interface because of the relatively poor strength, which led to debonding between the fibres and matrix. Besides, basalt fibres would fracture when the stress is beyond the strength. In order to investigate the influence of SMA fibres and different fibre surface modification in hybrid composite laminates, time-load, deflection-load and time-energy curves are plotted in Figure 12, Figure 13 and Figure 14 to illustrate the performance of different samples.

In Figure 12, it can be clearly seen that all the specimens embedded with SMA fibres have higher maximum contact load comparing to specimen without SMA fibres. However, specimen with unmodified SMA fibres only have limited enhancement comparing to specimen without SMA fibres. Sample modified with both KH550 and Al_2_O_3_ nanoparticles have higher maximum contact load than other samples, which proves that specimen treated with both KH550 and Al_2_O_3_ nanoparticles have the best damage tolerance among all the SMAHC laminates [10]. In Figure 12, it can be seen that the time-contact load curves of specimens without SMA fibres and embedded with etched SMA fibres show typical two-peak shape while the decreasing of contact load of specimens with untreated SMA fibres and SMA fibres modified with KH550 is more severely than other samples. Previous study [49] pointed out that the sudden drop on time-load curves indicate damage in composite laminates. The sudden decreasing of contact load of specimens with untreated SMA fibres and SMA fibres modified with KH550 can be attributed to the debonding between SMA fibres and polymer matrix. Although the SMA fibre modified with KH550 have relatively good bonding performance, when the maximum cohesive force is reached, debonding will evolve more rapidly comparing to other samples, which also can be observed in single fibre pull-out experiment [43]. To sample modified with both KH550 and Al_2_O_3_ nanoparticles, the drop of contact load is not as severe as other samples, which indicate that there is less damage in the sample. In other words, nanoparticles on the surface of SMA fibres can reduce the damage evolution along the interface between SMA fibres and polymer matrix. The maximum contact load of different specimens are listed in Table 2. Samples modified with both KH550 and Al_2_O_3_ nanoparticles have the highest contact force among all the samples. In Figure 13, it can be seen that the specimen without SMA fibres have larger deformation than samples embedded with SMA fibres. Besides, specimens with unmodified fibres have the largest deflection among all the specimens embedded with SMA fibres. The larger deflection of specimen embedded with unmodified SMA fibre is caused by the lower stiffness, leading to the lower contact force during impact process. The energy of the different specimens is presented in Figure 14. Rebound energy and absorbed energy are marked with different colour in Figure 14 according to previous research [50]. It can be seen that all the specimens with SMA fibres absorb more energy than that without SMA fibres while the rebound energy of sample without SMA fibres is higher than other samples. Under low-velocity impact, SMA fibres in composite laminates underwent a stress-induced martensite (SIM) process, which is associated with energy absorption [50]. All the specimens embedded with SMA fibres have similar energy absorption capacity, which indicate that the SMA surface modification have limited influence to the energy absorption performance of composite laminates. From the results, it can be concluded that embedding SMA fibres in BFRP composites can significantly enhance the impact resistance capacity of the composite laminates. Surface modification of SMA fibres can improve the performance of the interface between the SMA fibres and polymer matrix, which can further enhance the impact resistance performance of the composite laminates.

## 4. Conclusions

In this paper, the influence of embedded SMA fibres and SMA surface modification were studied through tensile, three point bending and low velocity impact tests.

The following conclusions can be drawn:

- The SEM images illustrated that the surface roughness of SMA fibres were enhanced by etching with H_2_SO_4_ and NaOH. The fibre surface modified with KH550 have no significant different from etched SMA fibre. After modified with both KH550 and Al_2_O_3_ nanoparticles, well dispersed nanoparticle can be found on the fibre surface, which significantly enhance the fibre surface roughness. After impact tests, it can be found that there are more polymer matrix adhered on the debonding SMA fibres, especially that modified with KH550 and both KH550 and Al_2_O_3_ nanoparticles, which prove the surface treatment can enhance the bonding between SMA fibres and polymer matrix.
- In three point bending tests, specimens embedded with SMA fibres that were unmodified, corroded with H_2_SO_4_ and NaOH solution, modified by the silane coupling agent KH550, or modified by both the silane coupling agent KH550 and Al_2_O_3_ nanoparticles have a 2.66%, 5.10%, 8.43% and 10.91% enhancement in the average ultimate tensile strength, respectively, compared to the BFRP specimen without SMA fibres. The new modification method with both the silane coupling agent KH550 and Al_2_O_3_ nanoparticles show the best effect among all treatment methods because the interface roughness and chemical bonding between the Ni-Ti fibres and polymer matrix were both improved.
- The samples with embedded SMA fibres that were unmodified, corroded with H_2_SO_4_ and NaOH solution, modified by the silane coupling agent KH550, or modified by both the silane coupling agent KH550 and Al_2_O_3_ nanoparticles have a 12.60%, 24.02%, 30.02% and 40.31% enhancement in the average flexural strength, respectively, compared to the BFRP specimen without SMA fibres. Embedding SMA fibres in the BFRP composite laminate have better enhancement effects on the specimens for the three-point bending test than on the specimens for the tensile test, which can be attributed to the location of the SMA fibres in the bending-test specimens. SMA fibres located in the lower part of the specimens are exposed to larger stress, which better exploits the performance of Ni-Ti fibres.
- In low-velocity impact test, specimens embedded with SMA fibres unmodified, corroded with H_2_SO_4_ and NaOH solution, modified by silane coupling agent KH550, modified with both silane coupling agent KH550 and Al_2_O_3_ nanoparticles have 5.11%, 16.55%, 19.22% and 24.33% enhancement in contact force, respectively, comparing to BFRP specimens without SMA fibres. Samples embedded with SMA fibres also have better energy absorption performance, which is induced by the SIM property of SMA fibres. Modification with both KH550 and Al_2_O_3_ nanoparticles also have the best effect among all the treatment methods.

## Figures and Tables

**Table 1:** Brief summary of different specimens.

Group of Specimens	SMA Treatment Methods	Specimen Types	Dimension	Content of Basalt Fibres (vol %)	Number of SMA Fibres
1	Without SMA fibres	Tensile	200 × 25 × 1.7 mm	50.76	^__^
		Three-point bending	69 × 13 × 1.8 mm	47.94	^__^
		Impact	100 × 100 × 1.8 mm	47.94	^__^
2	Without treatment	Tensile	200 × 25 × 1.7 mm	50.76	10
		Three-point bending	69 × 13 × 1.8 mm	47.94	5
		Impact	100 × 100 × 1.8 mm	47.94	13
3	Etched with H_2_SO_4_ and NaOH	Tensile	200 × 25 × 1.7 mm	50.76	10
		Three-point bending	69 × 13 × 1.8 mm	47.94	5
		Impact	100 × 100 × 1.8 mm	47.94	13
4	KH550	Tensile	200 × 25 × 1.7 mm	50.76	10
		Three-point bending	69 × 13 × 1.8 mm	47.94	5
		Impact	100 × 100 × 1.8 mm	47.94	13
5	KH550 and Al_2_O_3_ nanoparticles	Tensile	200 × 25 × 1.7 mm	50.76	10
		Three-point bending	69 × 13 × 1.8 mm	47.94	5
		Impact	100 × 100 × 1.8 mm	47.94	13

**Table 2:** Maximum contact load of specimens in the low-velocity impact test.

Specimen	Max. Contact Load/kN	Increase Ratio/%
Laminate without SMA fibres	4.11	^__^
Laminate with untreated SMA fibres	4.32	5.11
Laminate with etched SMA fibres	4.79	16.55
Laminate with SMA fibres modified with KH550	4.90	19.22
Laminate with SMA fibres modified with KH550 and Al_2_O_3_ nanoparticles	5.11	24.33
